# Bibliographical Notices

**Published:** 1847-04

**Authors:** 


### BIBLIOGRAPHICAL NOTICES.

*A Dictionary of Practical Medicine: comprising General Pathology, the Nature and Treatment of Diseases, Morbid Structures, and the Disorders especially incidental to Climates, to the Sex, and to the different Epochs of Life; with numerous prescriptions for the Medicines recommended, a classification of Diseases according to pathological principles, a copious Biography, with references, and an appendix of approved Formulæ: the whole forming a Library of Pathology and Practical Medicine, and a Digest of Medical Literature.* By James Copland, M. D., F. R. S., Fellow of the Royal College of Physicians; Honorary Member of the American Philosophical Society, &c. Part XI. London: 1847.

Another part of this valuable Dictionary, which "drags its slow length along," has just reached us from England; comprising the remainder of the article on "*hæmagastric pestilence,*" (yellow fever,) *septic or glandular pestilence* [a bad name] or plague; *protection from pestilences; phlegmasia alba dolens; Pityriasis* and *Diseases of the pleura*, (in part.) It bears all the wonted characteristics of the Author's ability and industry; but exhibits less freedom from emotion, and more unilateral prepossessions than many—perhaps any—of its predecessors. We notice it especially to draw the attention of the able American editor to the exceptionable tone, and imperfect and unsatisfactory exposition of the different views advocated on the great question of communicability of yellow fever; and to the general neglect of facts and opinions entertained on the subject on this side of the Atlantic, except where they accord with those of the author,—who is an ultra-contagionist. The tone in which he supports his views may be appreciated by the following extract:

"It cannot be denied by any one who has attended to the subject of quarantine, especially as it has been agitated in recent times, and with a due knowledge of the influence which the ruling passion—the desire of amassing wealth—exerts upon all the more generous and social emotions of the mind, that the restrictions imposed upon trade, arising out of precautions against the introduction of pestilential infections, have been the chief causes, directly or indirectly, producing the opposition to the doctrine of the infectious properties of pestilences; and that all that has been written to disprove this doctrine—and written with no small virulence by some—has not proceeded from a firm conviction of the justice of the cause espoused, but are either special pleadings subservient to sordid purposes, and to the gratification of disappointed feelings or of private resentments, or the outpourings of minds teeming with mistaken views, arising out of imperfect observation and hastily formed opinions, and excited by a desire of acquiring notoriety in a contest involving the interests of the whole community." p. 152.

Nothing can well be more objectionable than the tone of the above paragraph, as applied to a matter of scientific investigation and discussion. In this country, the mass of observers have arrived at conclusions diametrically opposed to those of the Author; and if they were to forget proprieties, they might with as much foundation hurl back the charges of interestedness, &c., upon him.

His next paragraph in continuation of the subject is not less objectionable.

"Let any one altogether unprejudiced as to the infectious or contagious properties of pestilential maladies, attentively peruse most of what has been written respecting them in this and in other countries, carefully examine the evidence adduced before committees of the House of Commons, or in other places, and critically weigh the import and truth of the conclusions arrived at by commissions sent to investigate facts on the spots of their occurrence, and the various circumstances connected with the facts adduced—let any one who possesses sound common sense, with some share of science, but who is at the same time entirely free from the undue influence of prejudice, of temper and of interest, enquire into the matter—and I cannot believe that he can arrive at a different conclusion from that to which I have arrived, after the best attention I have been able to bestow upon this most important and much discussed subject. Whoever may enter upon this very unpleasant investigation, with these moderate qualifications, which, however necessary, are quite sufficient to the formation of just conclusions respecting it, will be surprised to find that, amongst members of a learned profession, so much ignorance should be displayed in the literary character of some of these writings, in the scientific and professional execution of others, and in the illogical inferences of many of them. The duly qualified and candid investigator will detect statements made without proof, facts assumed without evidence, and suppositious agents believed in as real existences, and these made the bases of reasonings altogether inconclusive even as regards the conduct of the argument. He will find things, facts and diseases dissimilar from one another, and presenting no connection either as to nature or to sequence, viewed as identical with each other. He will detect the suppression of important facts and circumstances, and an undue prominence given to others of a doubtful character. He will remark the imputation of motives which did not exist, and ignorance of those which influenced, if they did not impel the writers. He will observe the precipitancy with which the young, the inexperienced and the ignorant, have rushed into print, and attacked with disgusting flippancy and intemperance much abler and better informed writers. In every medical periodical existing during the late war, he will find accounts of a disease never seen by the describers, their own mistakes, proceeding from profound ignorance of the name and nature of the malady seen by them, serving as the basis of their lucubrations and their arguments. And he will, moreover, be grieved to remark the opinions of learned and experienced men either misrepresented or impugned in jejune and paltry performances, evincing a most remarkable ignorance of the language in which they are written, and a still greater ignorance of that from which they profusely and inappropriately quote. In thus attempting to reach the pure spring of truth at the bottom of the deep code of research, he will have to penetrate not only through the rubbish thrown in by unfaithful, by mistaken, and by ignorant inquirers, but also through the accumulated filth of uncandid and intemperate controversy."

This is a strange exordium for one whose mind has been made up on one side; and who is assuredly most sparing in his references to testimony on the opposite view of the question,—a view, too, which is embraced on this side of the Atlantic by most of those—and many of them highly distinguished in their profession—who had ample opportunities for investigating the whole matter during the prevalence of yellow fever epidemics, in Philadelphia more especially; not one of whom—we are persuaded—was in the slightest degree swayed by the unworthy motives ascribed to supporters of that side of the question by the author of the article before us.

What greater intolerance could they have exhibited than that which is comprised in the above extract, or in the following?

"The opinions of several physicians are adduced by Dr. Bancroft in favour of the non-infectious nature of this pestilence; but upon referring to them, it will be found that they actually support a very opposite doctrine; and that their ideas, as to a non-infectious character, had reference entirely to the remittent endemics of which they were treating, and not to epidemic yellow fever—a piece of sophistry of the most dishonest and contemptible kind."

We hope that the American Editor of the Dictionary—Dr. Lee—will be full in his citations of authorities on both sides of this vexed question, in order that the article may be truly cyclopædiac in its character, and more worthy of companionship with the many admirable essays contained in the work.

It is long since Part X appeared; and if the remaining parts are as tardy in presenting themselves, it must be many years before the Dictionary is completed.

*Cours de Microscopie Complémentaire des Etudes Médicales Anatomie Microscopique et Physiologie des Fluides de l'economie. Atlas exécuté d'après Nature au Microscope Daguerréotype.* Par M. Donné, Docteur en Médecine, ex-chef de Clinique de la Faculté de Paris, Professeur particulier de Microscopie, &c., et Léon Foucault. Fol. p. 30. Paris, 1845-1846.

The text of the *Cours de Microscopie* of M. Donné was published in 1844, in Paris, but the Atlas before us has only just been completed. M. Donné has long been celebrated for his chemical and other investigations into the nature of certain of the animal secretions, and especially of the milk. His *Mémoire sur le Lait* was published many years ago, and has been exhausted—he informs us;—and it was in consequence of his having been invited to issue a new edition of it, that he determined to reproduce it in a separate work, and to associate with it his microscopic researches into the different fluids of the economy. Accordingly, the *Cours de Microscopie* contains the whole of his examinations of mucus, urine, sperm, milk, &c.

"These researches"—he observes—"extended and perfected as far as it was practicable for me since my first publications, united with the physiological considerations and practical applications that flow from them, form the principal portion of the lectures which I now reproduce." p. 9.

In his introduction to the "*Cours*," M. Donné announced, that an Atlas would be added to the work, and that it would present an innovation. "It will comprise," he remarked, "figures of two orders—the one will be executed according to the ideas I have formed of the intimate structure of the microscopic objects depicted: these systematic figures are intended to elucidate the descriptions in the text, and to complete them. Alongside these figures will be placed others representing accurately the objects independently of all interpretation. To attain this result I have been desirous not to trust either my own hand or that of a designer, always more or less influenced by the theoretical ideas of the author. Profiting by the marvellous invention of the daguerreotype, the objects will be reproduced with a rigorous fidelity unknown hitherto by means of photographic processes." And he subsequently added :—

"The first trials which I made to apply the daguerrean method to the reproduction of microscopic objects will be recollected. Four years ago I had the honour to present to the Académie des Sciences a daguerreotype microscope, by means of which I had obtained the images of several objects of natural history, and of certain tissues, such as the osseous and the dental. Since then, these trials have been resumed by a young *savant*, a distinguished *amateur* of photography. The results obtained by M. Léon Foucault with the daguerreotype microscope, not only on solid objects, but on the intimate particles of fluids,—such as the blood corpuscles of different classes of animals, the globules of milk, mucus and pus, zoospermes, &c., are truly most remarkable, and all give a special value to our Atlas. Our collection of designs is not yet complete, but what we already possess permits us to announce to micrographers results altogether worthy of their attention and interest."

The atlas before us is the one promised in the "*Cours*," and certainly the promises are well fulfilled. The representations of the fluids microscopically examined are beautiful, and their truthfulness cannot admit of question. Portions of the objects seen in the field of the microscope have not been selected; the *whole* field is given as it presents itself to the eye of the observer, and hence a much more correct idea is formed of the object—than if—as in the case of the blood corpuscles—detached portions were selected.

The plates are twenty in number, and afford varied and well executed representations of the blood corpuscles of man and certain animals; the circulation of the blood in the frog's tongue; mucous globules; epidermic cells; vibratory cells of the mucous membrane; pus globules; crystallization of healthy saliva; crystals of cholesterine; globules of yeast, and of fermenting diabetic urine; crystals of nitrate of urea; the crystallization resulting from the evaporation of the urine of a patient affected with typhoid fever; crystals of uric acid; divisions of the micrometer; pulverulent crystallization of urate of ammonia from the urine; minute crystals of uric acid proceeding from the decomposition of urate of ammonia by acetic acid; crystals of oxalate of lime from the urine, and of ammoniaco-magnesian phosphate; white filaments, such as are found in the first drops of healthy urine at each emission; blood corpuscles presenting the annular form which they affect when in contact with urine; zoospermes of man and certain animals; crystallization of healthy urine evaporated on a plate of glass; ovulum of the rabbit in and out of the Graafian vesicle; ovula of the frog and salamander; globules of cow's milk; casein coagulated owing to the decomposition of milk and mixed with milk globules; globules of healthy human milk and of that of the ass and goat; the colostrum of the human female; milk of a woman delivered eight days, and not giving suck; muscæ volitantes; globules of potatoe starch; blood corpuscles of the salamander; and pollen grains of the flower of the mallow.

Great variety of illustration is presented; and both "Atlas" and "Cours" ought to be in the possession of every histologist and microscopist.

---

*Address to the Graduates of Geneva Medical College, deliverea January 26th,* 1847. By Charles Alfred Lee, A. M., M. D., Professor of General Pathology and Materia Medica in Geneva Medical College, etc. etc. Published by request of the Graduates.

This is an able address, and admirably suited to the occasion on which it was delivered. The valedictory address of a professor to his pupils, is almost necessarily monitory in its character. It is like the parting words of a parent to his son, pointing to his moral responsibilities and the great ethical rules by which he should be governed, and hence we rarely look for anything original, argumentative or ingenious; but it is something to express ordinary truths in language befitting the occasion, and calculated to win the attention, engage the affections, and persuade the judgment of the hearers. In these respects Dr. Lee has certainly been successful. We have room for only one or two extracts, which we select, not for any novelty in either the subjects or the sentiments, but for their importance and truthfulness.

"For some time after commencing your professional life, you will probably have some leisure time on your hands, which you can turn to profitable account by devoting it to study. Be not discouraged at the want of speedy success; your merits will eventually be known, and you will be rewarded accordingly. Justice will sooner or later be done you, and if you aim at eminence, and your efforts are well directed, you will attain it. Aim first at the establishment of character and reputation, with the full assurance that all desirable consequences will follow in their train.

Turn not aside into any of the devious, albeit fashionable, paths of quackery, so rife at the present day, by whatever specious name they may be known; sacrifice not your prospects and your good name by becoming the adherents of any partial and exclusive systems of medicine, for you may rest assured that they will all speedily disappear 'like the baseless fabric of a vision.' Belittle not your honorable title of PHYSICIAN by prefixing to it any distinctive or diminutive epithet, be it Thomsonian, Homœopathic, or Hydropathic; for why should you do this? Is not he who stands upon the broad platform of catholic medicine more likely to be better armed for attacking disease, than he who occupies some insignificant redoubt, or petty loop-hole. Is there any want of freedom of opinion in our profession? Is not every one at liberty to construct his own articles of faith, drawing from every system whatever portion of truth it may contain, and shape his practice accordingly? Medicine is not, as many seem to suppose, a system of rules and doctrines handed down from teacher to pupil, admitting no change, a set of formulæ which you are bound to sustain, and from which it were heresy to swerve; but it is a progressive and constantly improving science, and every true and sincere votary of it will employ *all* the remedies, means and resources within his reach, which accident or science has discovered, and observation and experiment verified. Away, then, with your partial systems which inevitably and professedly limit these means, and virtually nullify these resources. There is, indeed, Gentlemen, a sad relaxation of principle at the present day, even in some who are regarded as among the most distinguished members of the profession, as manifested by their patronage and recommendation of patented and secret remedies; a course of conduct which is obviously incompatible with every sentiment of moral duty, and every principle of sound medical ethics. To keep from the world any discovery calculated to benefit mankind, as connected with the preservation of human health, or its restoration when lost, is such a dereliction of duty, as to have met with the reprobation of the wise and good in every age of the world; and when this is done, as it generally is, for the sake of pecuniary emolument, the mind instinctively revolts at it, as an exhibition of selfishness and insensibility to human suffering disgraceful to our natures, and derogatory to the character of those who belong to a profession, whose foundation is philanthropy, and whose crowning glory is benevolence. Give not, then, the slightest encouragement to remedies of this description, or their inventors; frown indignantly upon all attempts to render our glorious art a mercenary trade; disgrace not your *Alma Mater* and your own reputation, by countenancing, in the slightest degree, any unworthy proceedings of this kind, for by so doing you will justly forfeit all title to respect, and take rank with the Brandreths and Moffats of the day. The time is not distant when such a deep stain of disgrace must inevitably attach to patentees and proprietors of secret remedies in our profession, that neither the waters of Lethe will be able to obliterate, nor the exhibition of 'Letheon' to bury in oblivion.

Should any of you, then, hereafter discover a remedy calculatep to benefit the world, publish it upon the house-top, imitate the goodness of Providence, and make it free as the air we breathe; for the consciousness of having done a good deed for humanity, the gratitude of an intelligent community, and the praises of a liberal profession, shall prove a most satisfactory reward.

There is one duty which you owe to yourselves, to the sick who may be entrusted to your charge, and to society, by whose favour and confidence you are to be sustained, and this is—to shun the use of intoxicating drinks. I urge this upon you, Graduates of Geneva College, by every consideration of duty, of honour, of interest, and of philanthropy; I charge you, as you value reputation, usefulness, success, and an approving conscience, ever adhere to the strictest rules of temperance. You owe this to those who have sustained you thus far, and furnished you with the means of obtaining a medical education; you owe it to your teachers who have labored to instruct you in the various branches of your art, and who feel an anxious desire for your prosperity; you owe it to the beloved Alma Mater, who sends you forth with pride this day, to carry health and virtue and gladness to those who come within your influence; you owe it to the profession whose bright escutcheon must never be soiled by your example; you owe it to society in whose ranks you are now to be enrolled, we trust among its most valued and respected members; you owe it to your hopes of usefulness here, and of happiness hereafter; you owe it to a reformed and enlightened public opinion; and, lastly, you owe it to your God. As you go forth, then, upon the serious errand of your lives—an errand requiring the keen eye, the cool head, the steady hand, the sound judgment—take this, my solemn and affectionate warning, along with you; carry it into the social circle and the recesses of private life; take it into the hospitable mansions of the rich and the lowly dwellings of the poor; remember it in the hour of temptation and trial; heed it when the syren voice of pleasure beckons you along her flowery paths; so shall your lives flow equably along; your cup of happiness be filled; your days crowned with usefulness, and your names with honour."

